# Enhanced Electrorheological Polishing Efficiency of Alumina-Doped Titanium Dioxide Particles

**DOI:** 10.3390/ma16062347

**Published:** 2023-03-15

**Authors:** Xufeng Hu, Han Sun, Xiaopeng Zhao, Jianbo Yin

**Affiliations:** 1 Research and Development Institute of Northwestern Polytechnical University in Shenzhen, Shenzhen 518057, China; 2 School of Physical Science and Technology, Northwestern Polytechnical University, Xi’an 710129, China

**Keywords:** ER polishing, alumina-doped titanium dioxide, *Surface quality*

## Abstract

Electrorheological (ER) polishing is a novel polishing technology having flexible and tunable characteristics. At present, ER polishing uses ER particles to drive abrasive particles to polish the material surface. Under the action of high-speed centrifugation, the abrasive particles are easily separated from ER particles due to their significantly different ER effect, and this can easily cause the degradation of polishing ability. In this work, alumina-doped titanium dioxide ER polishing particles were developed via a sol-gel method. As a classical abrasive, alumina has higher hardness and can improve the ER effect of titanium dioxide by doping. Thus, alumina-doped titanium dioxide particles simultaneously possess high ER effect and high hardness. No phase separation appears in the polishing process and the result shows that alumina-doped titanium dioxide has a good polishing efficiency for materials with Mohs hardness of 3 and below.

## 1. Introduction

ER fluid is a kind of intelligent suspension [1]. It consists of dielectric particles and insulating liquid. Dielectric particles can change their state in insulating liquid as the external electric field changes [2]. Thus, the suspension can be converted between Newtonian fluids and Bingham fluids [3]. ER materials mainly include inorganic substances [4], organic polymers [5] and composite materials [6]. Because the solid–liquid transformation of the ER fluid is quick, reversible, and adjustable, ER fluid has been used in many fields, such as polishing, clutch, sensor, artificial muscle, ink, etc. [7,8,9,10,11,12,13].

ER polishing is a novel polishing technology. Since Akagami et al. first reported the use of particle dispersed ER fluid for polishing [14], ER polishing has been widely studied because of its flexibility and controllability. In 2010, Hung et al. polished SKD11 using ER polishing technique. By comparing the influence of different electrode cutter sizes on the polishing effect, they found that the polishing effect of the large-size electrode cutter was lower than that of small-size electrode cutter. The reason for this might be that it was difficult for large-sized polishing tools to maintain good parallelism with the workpiece, and the surface of the workpiece was unevenly stressed, resulting in different surface quality of the workpiece [15]. Later, Zhang et al. designed a five-axis linkage ER polishing equipment [16]. Chen et al. proposed a single-side needle-tube electrode, where the needle was positive but the tube was negative [17]. Su et al. improved the surface quality of optical glass using a wheel-shaped ER polishing electrode tool [18]. Yang et al. found that ER polishing technology could remove directional texture and inhibit dispersion of optical elements effectively [19]. In addition, ER polishing was also combined with other polishing techniques to achieve a more efficient finishing. For example, Liu et al. used the ER effect to assist ultrasonic polishing in 2022 [20]. The polishing particles were better concentrated in the polished area with the assistance of ER fluid in the circumstances. Chen et al. combined ER polishing with magnetorheological polishing and prepared a particle that could respond to both electric and magnetic fields. The efficiency of electromagnetic rheological composite polishing was higher than that of single magnetic field polishing [21]. Although the ER polishing technique exhibits many advantages, it is difficult to apply ER polishing to actual machining. At present, ER polishing uses ER particles to drive abrasive particles to polish the material surface. The polarization properties and gravity of ER particles are completely different from abrasive particles. Abrasive particles easily break away from the bondage of ER particles to form another phase under the action of high-speed centrifugation. The removal of abrasive particles from the ER fluid leads to the deterioration of polishing properties in some areas, and finally makes the polishing effect unstable.

To eliminate this phase separation phenomenon, we recently developed composite ER polishing particles by Pickering emulsion polymerization. Alumina nanoparticles were embedded on polymerized ionic liquid particles with ER activity by Pickering emulsion polymerization [22]. Phase separation problems did not exist during polishing with composite particle ER polishing fluid. Thus, it showed good polishing performance. In addition, to improve polishing performance, we also developed a kind of cerium-doped titanium dioxide ER polishing particles. The particles were prepared by the sol-gel method and calcined at 500 °C. It has excellent ER properties and certain hardness. The shear stress of ceria-doped titanium dioxide ER polishing fluid could reach 1.1 kPa (3 kV/mm, 1 s^−1^). Compared with simple mixture of ceria and titanium dioxide, the phase separation problem could also be solved by ceria-doped titanium dioxide ER polishing fluid. The ceria-doped titanium dioxide ER polishing fluid showed better polishing property [23]. Due to low particle hardness, however, the polishing efficiency of the composite polishing fluids based on alumina-coated polymerized ionic liquid particles and cerium-doped titanium dioxide still needs to be improved. Doping is an important method to regulate the properties of substances. The introduction of impurities has a special effect on pure substances. In particular, Al^3+^ play an important role in the capture of electrons and holes in crystals and the blocking of the recombination of electrons and hole pairs in titanium dioxide [24]. The presence of electrons and holes in the crystal is beneficial to its ER properties. However, the application of alumina-doped titanium dioxide particles in ER polishing has not been studied.

In this paper, alumina-doped titanium dioxide particles were developed as an ER polishing material. As a classical abrasive, alumina has higher hardness. Alumina-doped titanium dioxide particles will have good ER properties and hardness. The preparation process and structure characteristics of alumina-doped titanium dioxide particles were presented. The ER property of alumina-doped titanium dioxide ER polishing fluid was measured. The polishing performance of alumina-doped titanium dioxide ER polishing fluid were tested by polishing materials with different hardness. The reasons for the enhanced ER polishing efficiency of alumina-doped titanium dioxide particles are discussed.

## 2. Experimental Section

### 2.1. Preparation of Alumina-Doped Titanium Dioxide Particles

The preparation process of alumina-doped titanium dioxide particles with Al/Ti ratio of 0.1 is as follows. First, 20mL anhydrous ethanol was divided evenly into two beakers. 7.521 g Butyl titanate (TBT, Tianjin Kermeo Chemical Reagent Co., Ltd., Tianjin, China) was added to the beaker, 0.294 g aluminum chloride (AlCl_3_, Tianjin Damao Chemical Reagent Factory Co., Ltd., Tianjin, China) and 0.5967 g deionized water were dissolved into the other. The solution in the two beakers was shaken violently for 20 min before being mixed together. The mixed solution was stirred continuously for 3 h and left to rest overnight. The solution undergone the gel process at an atmosphere of 40 °C. The transparent gel was obtained after 4 days. The remaining moisture in the gel was removed at 120 °C. Precursor particles were obtained by grinding and screening. Finally, the precursor particles were calcined at 200 °C, 400 °C, and 500 °C, respectively, for two hours to obtain white alumina-doped titanium dioxide particles.

### 2.2. Preparation of ER Polishing Fluid

Alumina-doped titanium dioxide particles were dried prior to the configuration of ER fluid. The residual moisture in the particles was removed after vacuum drying at 120 °C for 12 h. The dried alumina-doped titanium dioxide particles were dispersed into silicone oil (50 cSt), and after stirring and ultrasonic dispersion treatment, ER polishing fluid (Volume fraction φ = 20%) was obtained. The density of alumina-doped titanium dioxide particles was 3.8 g/cm^3^ as measured by pycnometer method.

### 2.3. Characterization

The crystal structure of alumina-doped titanium dioxide was recorded by XRD (D2 PHASER X, Bruker Corporation, Germany). The shape and size of alumina-doped titanium dioxide were analyzed by SEM (Verios G4, Thermo Fisher Scientific, Waltham, MA, USA) and laser particle size analyzer (Nano-ZS90, Malvern Panalytical, Malvern, UK). The distribution of elements in alumina-doped titanium dioxide particles was characterized by EDS (Thermo NS7, Thermo Fisher Scientific, USA).

### 2.4. ER Properties and Polishing Properties Measurements

The ER properties were represented by shear stress-shear rate curves at different electric field intensities. Shear stress values at different shear rates were measured by a stress-controlled rheometer (HAAKE RS600, Thermo Electron (Karlsruhe) GmbH, Karlsruhe, Germany). The spacing of parallel plates (diameter 35 mm) in the system was controlled at 1 mm. The power supply used for the test was DC. The rheological properties were tested in CR mode (0.01–1000 s^−1^). The test voltages were 0, 0.5 kV, 1 kV, 2 kV, and 3 kV. The test temperature was room temperature. The fluid was restored to its initial state by pre-shearing before each test.

**Figure 1:**
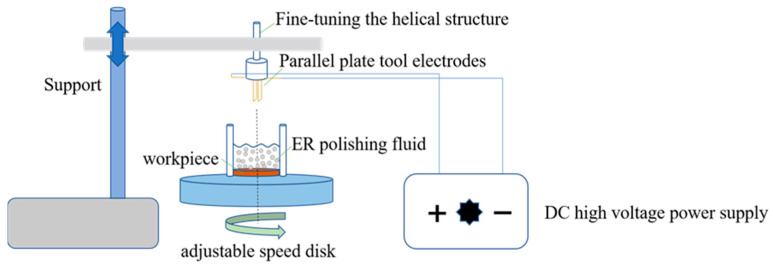
Structure diagram of ER polishing device.

Polishing properties were tested using a homemade ER polishing system, as shown in Figure 1. The tool electrode used in the polishing experiment was a single-sided electrode of a parallel plate that combines the positive and negative electrodes. The power supply used in the polishing experiment was DC. The voltage was adjusted in 0–4 kV. Polishing speed was 200 r/min. The machining gap could be coarsely adjusted by lifting and lowering the beam and fine-tuned by the spiral structure. The workpiece and the ER fluid were placed in an open container. The workpiece used in this article was copper (Mohs hardness = 1) and brass (Mohs hardness = 3).

The roughness correlation data for the workpiece were measured using a surface profilometer (SR2000, Shaanxi Weir Electromechanical Technology Co., Ltd., Xi’an, China). Because the yield surface roughness parameters of the workpiece we used are within the application range of Ra (0.008–400 μm, ISO468-82), we used Ra to indicate the surface finish of the workpiece. Ra is arithmetical mean deviation of the profile. The cut-off wavelength was selected as 0.08 mm and the evaluation length is 0.4 mm. The average roughness was obtained from 10 measured Ra. The surface texture of the brass was observed by reflection microscope (50×) (ECLIPSE MA100N, Nikon Corporation, Japan). The surface morphology of the brass workpiece was characterized by AFM (Bruck Dimension Icon, Bruker Corporation, Karlsruhe, Germany). The scanning area is 10 × 10 μm.

## 3. Results and Discussion

### 3.1. Material Structure

**Figure 2:**
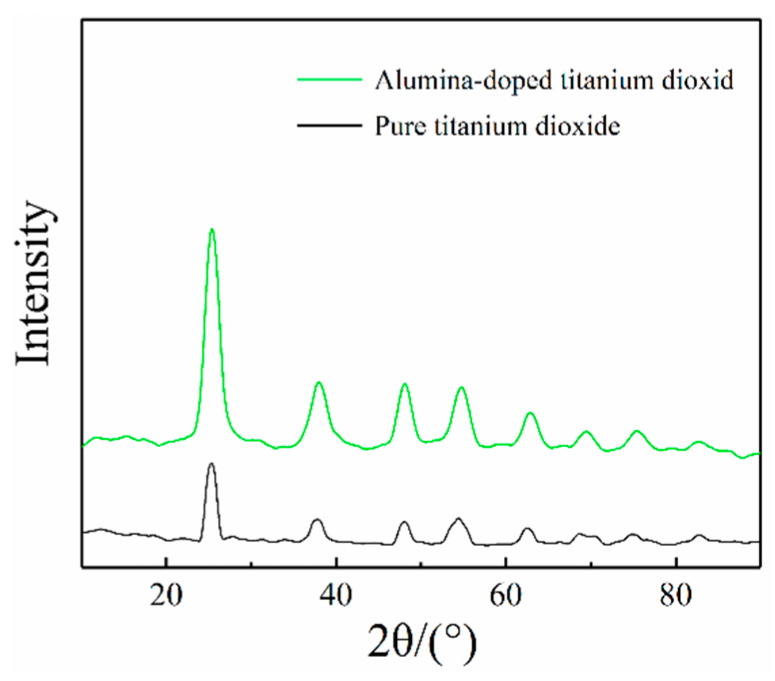
XRD pattern of alumina-doped titanium dioxide and pure titanium dioxide particles.

Figure 2 shows XRD pattern of pure titanium dioxide and alumina-doped titanium dioxide particles. The crystal structure of alumina-doped titanium dioxide particles prepared at 500 °C calcination temperature is anatase. From the point of view of the intensity of the diffraction peak, alumina-doped titanium dioxide has higher crystallinity, which is favorable to the hardness of alumina-doped titanium dioxide particles.

**Figure 3:**
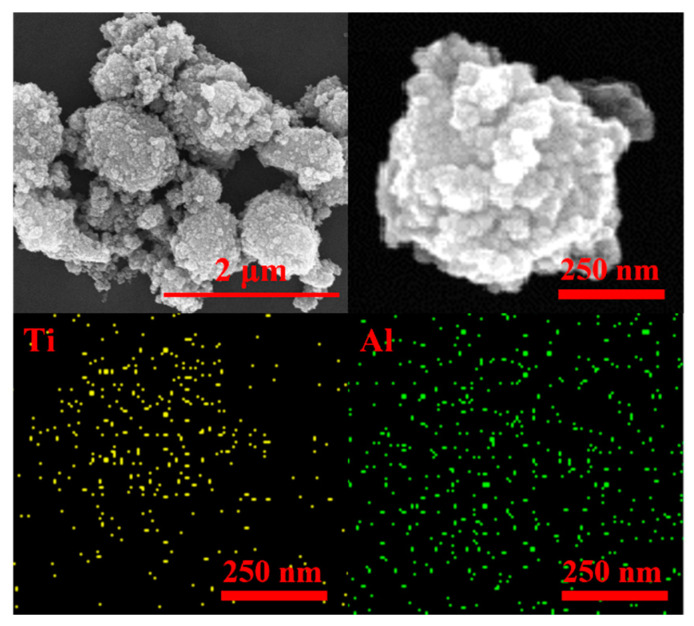
SEM images and EDS analysis of alumina-doped titanium dioxide particles.

Figure 3 shows the SEM image and distribution of element of alumina-doped titanium dioxide particles. The shape of alumina-doped titanium dioxide particles prepared by sol-gel method and calcined at high temperature is irregular. Its particle size is not uniform, both micron scale large particles and nano-scale small particles coexist. The sharp large particles are conducive to cutting the workpiece surface, and small particles are conducive to obtaining high surface quality. By analyzing the EDS, it can be concluded that alumina is uniformly doped in titanium dioxide particles.

**Figure 4:**
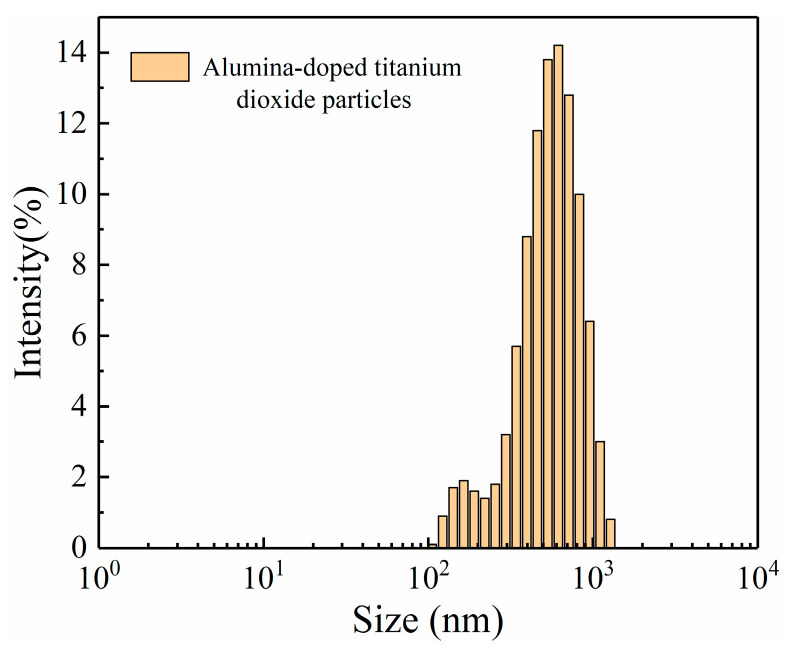
Size distribution of alumina-doped titanium dioxide particles.

Figure 4 shows the particle size distribution of alumina-doped titanium dioxide particles. The size of alumina-doped titanium dioxide particles prepared at 500 °C calcination temperature is 105–1280 nm. This is consistent with the results observed by SEM. 

### 3.2. ER Property

**Figure 5:**
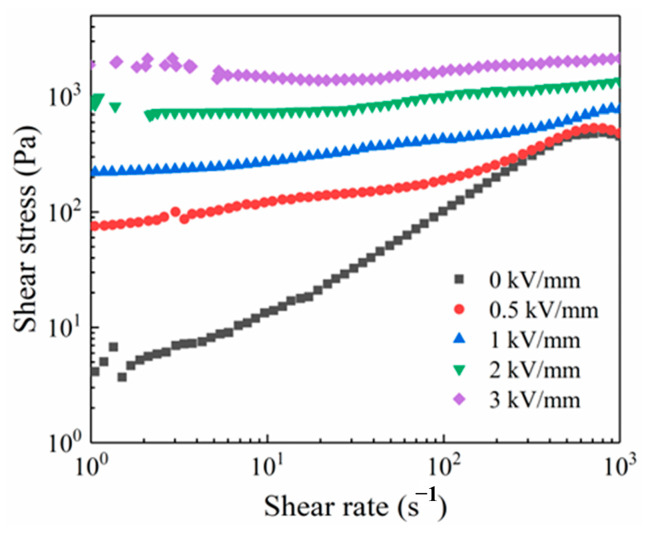
Rheological curve of alumina-doped titanium dioxide ER polishing fluid under different electric field intensity (volume fraction = 20%, T = 25 °C).

Figure 5 shows the rheological curve of alumina-doped titanium dioxide ER polishing fluid (20%) under different electric field intensity. The shear stress of alumina-doped titanium dioxide ER polishing fluid increases steadily with the acceleration of shear rate when there is no electric field. However, when the shear rate exceeds 700 s^−1^, there is an expulsion of the fluid from the gap of two electrodes, which leads to a slowdown or even a decrease in the shear stress due to the centrifugal force. However, with the increase of the electric field intensity, this phenomenon of shear stress reduction disappears, and the shear stress-shear rate curve presents a “plateau”. This is because the fluid exhibits the Bingham fluid state when it is exposed to an electric field, and its shear stress increase with enhancement of electric field intensity. At the same time, the force between the ER particles is stronger than the centrifugal force at high shear rate. As a result, no expulsion of the fluid from the gap of two electrodes appears, and the shear stress no longer increases with increasing shear rate. 

**Figure 6:**
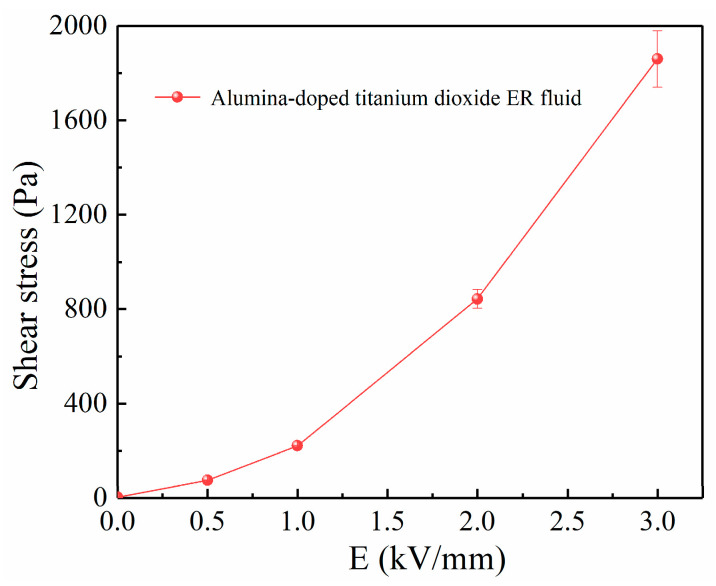
Shear stress as a function of electric field intensity for alumina-doped titanium dioxide ER polishing fluid (volume fraction = 20%, shear rate = 1 s^−1^).

Figure 6 shows the change of shear stress value of alumina-doped titanium dioxide ER polishing fluid (20%) with electric field intensity when shear rate is 1 s^−1^. The shear stress of alumina-doped titanium dioxide ER polishing fluid increases with the enhancement of electric field intensity. The ER effect is particularly evident at strong electric fields. The value of shear stress of alumina-doped titanium dioxide ER polishing fluid is 1.8 kPa at 3 kV/mm, while that of pure titanium dioxide ER polishing fluid is 400 Pa, and that of ER polishing fluid of simple mixture of titanium dioxide and alumina is 380 Pa. Under the same shear rate, the value of shear stress of alumina-doped titanium dioxide ER polishing fluid is far higher that of pure titanium dioxide ER polishing fluid and ER polishing fluid of simple mixture of titanium dioxide as ER particles and alumina as abrasive particles. In addition, the value of shear stress of alumina-doped titanium dioxide ER polishing fluid is also much higher than that of ceria-doped titanium dioxide ER polishing fluid reported before [23]. Aluminum ions provide hetero-valent doping for titanium dioxide crystals. As mentioned in Section 1, the presence of aluminum ion can increase the defect concentration in the crystal. The increase of defect concentration is beneficial to the electrical polarization in the crystal. This is the reason why alumina-doped titanium dioxide particles have a stronger ER effect. Higher shear stress is beneficial for the improvement of polishing.

### 3.3. ER Polishing Performance

**Figure 7:**
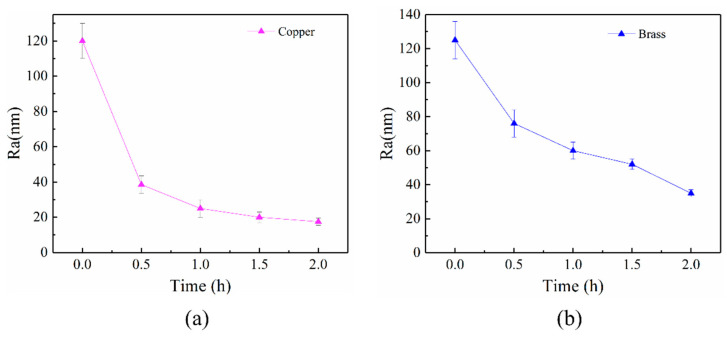
Surface roughness variation of copper workpiece (**a**) and brass workpiece (**b**) polished by alumina-doped titanium dioxide ER polishing fluid (rotation speed = 200 r/min, voltage = 3 kV, machining gap = 0.5 mm).

Figure 7 shows the variation of Ra values of copper workpiece surface and brass workpiece surface polished by alumina-doped titanium dioxide ER polishing fluid over time (rotation speed = 200 r/min, voltage = 3 kV, machining gap = 0.5 mm). With the change of polishing time, the surface quality of copper workpiece has been improved in different degrees. The initial Ra of copper workpiece surface is about 120 nm. The Ra is 38 nm after polishing for 0.5 h and below 20 nm after polishing for 2 h. However, the Ra of the copper workpiece surface does not present a significant decrease when the copper workpiece surface is polished for 0.5 h with pure titanium dioxide ER polishing fluid and with ER polishing fluid of simple mixture of titanium dioxide as ER particles and alumina as abrasive particles. In addition, we recently used ceria-doped titanium dioxide ER polishing fluid and the same experimental conditions to polish the same copper workpiece surface, and it was only able to reduce the Ra from 120 nm to 60 nm after 0.5 h of polishing [23].

On the other hand, the alumina-doped titanium dioxide ER polishing fluid can polish brass workpiece as shown in Figure 7b. The Ra of brass workpiece surface decreases from 126 nm to 31 nm after 2 h polishing under the identical experimental parameters as polishing copper workpiece. However, pure titanium dioxide ER polishing fluid and the ER polishing fluid of the simple mixture with titanium dioxide as ER particles and alumina as abrasive particles cannot polish the brass workpiece due to their weak ER effect. Although ER polishing fluid of ceria-doped titanium dioxide particles can polish the brass workpiece, but its polishing efficiency is much lower compared to alumina-doped titanium dioxide ER polishing fluid due to the relative low hardness. The above results indicate that the alumina-doped titanium dioxide ER polishing fluid can efficiently polish materials with hardness similar to brass. The high ER effect of alumina-doped titanium dioxide ER polishing fluid and high hardness of particles due to alumina doping are beneficial to the enhanced polishing efficiency.

**Figure 8:**
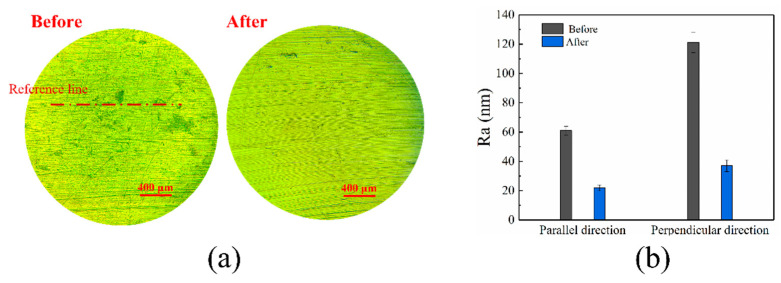
Surface morphology (**a**) and surface roughness (**b**) of brass workpiece polished for 2 h by alumina-doped titanium dioxide ER polishing fluid (rotation speed = 200 r/min, voltage = 3 kV, machining gap = 0.5 mm).

Figure 8 shows the surface morphology changes and the Ra values of brass workpiece surface before and after being polished by alumina-doped titanium dioxide ER polishing fluid. The surface morphologies of the brass workpiece before and after being polished were photographed by reflection microscope, as shown in Figure 8a. The unpolished brass surface has a large number of pits and scratches of uniform orientation and uneven thickness. The polished brass workpiece surface has obvious polishing marks, the polishing marks are uniform and flat, and the scratches and pits are reduced. We set the original traces of the brass workpiece surface as the reference line. For the brass workpiece surface, the roughness value is the highest in the direction perpendicular to the reference line, and the roughness value is the lowest in the direction parallel to the reference line. Therefore, if the roughness value perpendicular to the reference line decreases obviously after polishing, it indicates that the alumina-doped titanium dioxide ER polishing liquid has a good polishing effect on the brass workpiece surface. The surface roughness before and after being polished in the parallel and vertical directions was measured, as shown in Figure 8b. The Ra of the brass workpiece surface parallel to the original trace is reduced from 61 nm to 22 nm, and the roughness perpendicular to the original trace is reduced from 121 nm to 37 nm. The roughness of brass workpiece parallel to the reference line direction is lower than that perpendicular to the reference line direction before polishing. When testing along the direction perpendicular to the reference line, the surface roughness will be large due to the uneven scratch depth and spacing between scratches. The roughness of the polished brass surface parallel to the direction of reference line is close to that perpendicular to the direction of the reference line. This is because the polished brass workpiece surface is smooth with no noticeable height difference.

**Figure 9:**
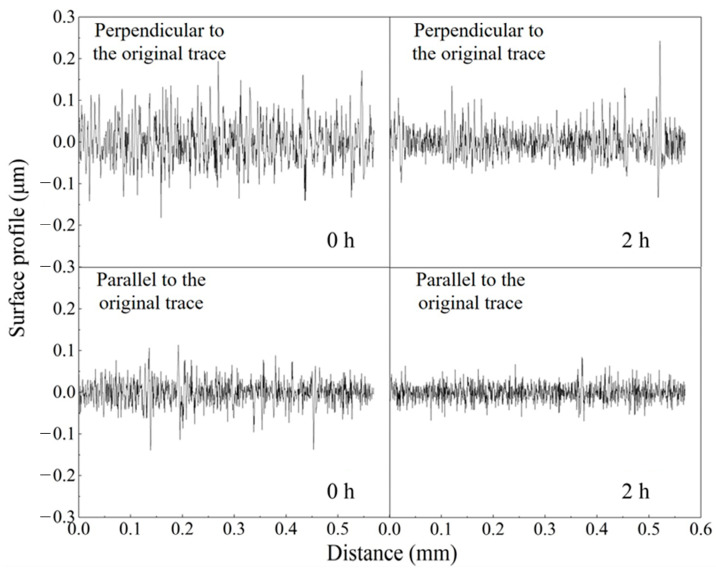
Surface profile curves of brass workpiece surface in parallel and vertical directions along the original marks before and after being polished with alumina-doped titanium dioxide ER polishing fluid (rotation speed = 200 r/min, voltage = 3 kV, machining gap = 0.5 mm).

Figure 9 shows the profile curves of the brass workpiece surface before and after polishing by alumina-doped titanium dioxide ER polishing fluid along the parallel and vertical directions of the original marks. As you can see, the fluctuation in the vertical direction is significantly larger than that in the parallel direction. However, the fluctuation in both directions after polishing is smaller than that before polishing. This indicates that the alumina-doped titanium dioxide ER polishing fluid has a good polishing effect for the brass workpiece with obvious original machining marks.

**Figure 10:**
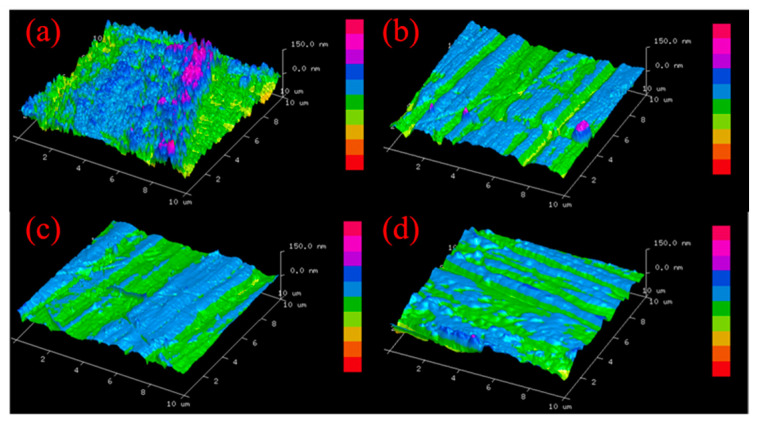
AFM image of unpolished brass workpiece surface (**a**) and three different positions of brass workpiece surfaces (**b**–**d**) polished for 2 h by alumina-doped titanium dioxide ER polishing fluid (rotation speed = 200 r/min, voltage = 3 kV, machining gap = 0.5 mm).

Figure 10 shows the AFM images of unpolished brass workpiece surface and three different positions on brass workpiece surface after polishing by alumina-doped titanium dioxide ER polishing fluid. The color in the Figure 10 is from red to purple, indicating that the roughness is from low to high. It can be seen that there are many bulges on the surface of the unpolished brass workpiece, as shown in Figure 10a. From the surface micromorphology of three different positions on the polished brass workpiece, it can be seen that the polishing marks of each polished area are clear, and their directions are different.

**Figure 11:**
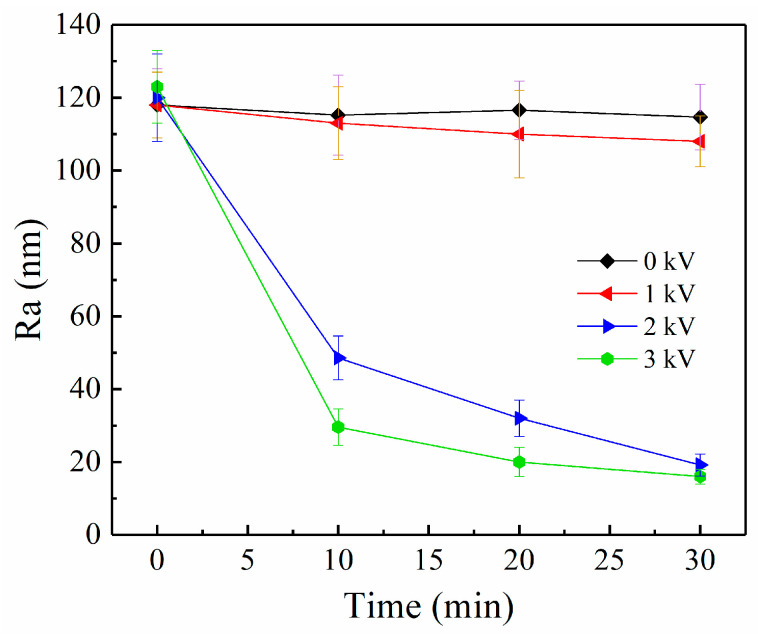
The roughness brass workpiece surface as a function polishing time at different working voltages (rotation speed = 200 r/min, machining gap = 0.2 mm).

Figure 11 shows the roughness change of brass workpiece surface with polishing time at different working voltages (rotation speed = 200 r/min and machining gap = 0.2 mm). When the voltage is 0 or 1 kV, the surface roughness of brass workpiece has little change. When the voltage is 2 kV and 3 kV, the surface roughness of brass workpiece decreases obviously in a short time and reaches 20 nm after 30 min of polishing. The ER polishing fluid has higher polishing efficiency at the voltage of 3 kV. In the absence of voltage, the interaction force between the polishing particles and the brass workpiece surface can be ignored because the polishing particles lack the contact pressure and cannot gather in the area to be polished due to centrifugal force. When the electric field is present, a polishing medium layer formed by the polarization of the ER polishing particles fills the machining gap. When the electric field intensity is weak due to the low voltage, the polishing medium layer formed by the ER polishing particles is not strong enough to effectively polish the brass workpiece surface. However, with the increase of voltage, the strength of polishing medium layer is enhanced. As a result, the ER polishing particles can achieve effective the cutting of the brass workpiece surface hump. 

## 4. Conclusions

In this paper, alumina-doped titanium dioxide particles were developed as an ER polishing material. The ER effect of alumina-doped titanium dioxide particles and the polishing performance was studied. Thus, it was found that the shear stress of alumina-doped titanium dioxide ER polishing fluid (20%) could reach 1.8 kPa when the electric field was 3 kV/mm and the shear rate was 1 s^−1^. The shear stress value was nearly 1.4 kPa higher than that of pure titanium dioxide ER polishing fluid and an ER polishing fluid of simple mixture with titanium dioxide as ER particles and alumina as abrasive particles, and nearly 700 Pa higher than that of ceria-doped titanium dioxide ER polishing fluid. Under the same polishing conditions (rotation speed = 200 r/min, machining gap = 0.5 mm, voltage = 3 kV, polishing time = 0.5 h), the Ra of copper workpiece surface polished with alumina-doped titanium dioxide ER polishing fluid was 22 nm, which was lower than that polished with ceria-doped titanium dioxide ER polishing fluid. Alumina-doped titanium dioxide ER polishing fluid could well polish the brass workpiece with higher hardness. The surface roughness of the brass workpiece could be decreased to 19 nm (Ra) after 30 min polishing by alumina-doped titanium dioxide ER polishing fluid under certain experimental parameters (rotation speed = 200 r/min, machining gap = 0.2 mm, voltage = 3 kV).

## Data Availability

Not applicable.
